# Fibroblast growth factor receptor 3 (FGFR3) aberrations in muscle-invasive urothelial carcinoma

**DOI:** 10.1186/s12894-018-0380-1

**Published:** 2018-07-31

**Authors:** Young Saing Kim, Kyung Kim, Ghee-Young Kwon, Su Jin Lee, Se Hoon Park

**Affiliations:** 1 Division of Medical Oncology, Department of Internal Medicine, Gil Medical Center, Gachon University College of Medicine, Incheon, South Korea; 2 Division of Hematology-Oncology, Department of Medicine, Sungkyunkwan University Samsung Medical Center, Sungkyunkwan University School of Medicine, 81 Irwon-ro, Gangnam-gu, Seoul, 06351 South Korea; 3 Department of Pathology and Translational Genomics, Sungkyunkwan University Samsung Medical Center, Seoul, South Korea

**Keywords:** Urothelial carcinoma, FGFR, Mutation, Fusion

## Abstract

**Background:**

Recent studies suggest that *FGFR3* is a potential therapeutic target in urothelial carcinoma (UC). The purpose of this study was to evaluate the rates and types of *FGFR3* aberrations in patients with muscle-invasive UC who received radical resection.

**Methods:**

We analyzed surgical tumor samples from 74 UC patients who had received radical cystectomy (*n* = 40) or ureteronephrectomy (*n* = 34). Ion AmpliSeq Cancer Hotspot Panel v2 and nCounter Copy Number Variation Assay were used to detect *FGFR3* aberrations.

**Results:**

Fifty-four patients (73%) had high-grade tumors, and 62% had lymph node involvement. Sixteen patients (22%) harbored *FGFR3* alterations, the most common of which was *FGFR3* mutations (*n* = 13): Y373C (*n* = 3), N532D (*n* = 3), R248C (*n* = 2), S249C (*n* = 1), G370C (*n* = 1), S657S (*n* = 1), A797P (*n* = 1), and 746_747insG (*n* = 1). Three additional patients had a *FGFR3-TACC3* rearrangement. The frequency of *FGFR3* aberrations was higher in bladder UC (25%) than in UC of the renal pelvis and ureter (18%) but the difference was not statistically significant (*P* = 0.444). Genes that were co-aberrant with *FGFR3* included *APC* (88%), *PDGFRA* (81%), *RET* (69%), and *TP53* (69%).

**Conclusions:**

We report the frequency and types of FGFR3 aberrations in Korean patients with UC. Patients with *FGFR3* mutations or *FGFR3-TACC3* fusion may constitute potential candidates for a novel FGFR-targeted therapy in the perioperative setting.

**Electronic supplementary material:**

The online version of this article (10.1186/s12894-018-0380-1) contains supplementary material, which is available to authorized users.

## Background

Urothelial carcinoma (UC), a cancer involving the transitional epithelium of the urinary tract, is the seventh most common malignancy in Korea [1]. The majority of cases arises in the bladder, whereas only about 5 to 10% occurs in the upper urinary tract including the renal pelvis and ureter [2]. Because of the relative rarity of upper tract urothelial carcinoma (UTUC), clinical decision making for patients with UTUC depends on data available for urinary bladder urothelial carcinoma (UBUC) [3].

For metastatic or advanced UC, platinum-based chemotherapy is considered standard treatment. There is a need to develop new therapeutic options focused on the molecular aberrations driving UC, as patients who fail to respond or have progressed after platinum-based chemotherapy have a grim prognosis. Recently, molecular analysis has identified subsets of UC expressing distinct molecular signatures. Genomic alterations in the fibroblast growth factor receptor 3 (*FGFR3*) are well described in UC and have led to extensive clinical investigations evaluating FGFR3 inhibitors [4]. FGFR3, which belongs to the family of tyrosine kinase, is responsible for the FGF signal transduction. FGFR3 signaling is involved in development, differentiation, cell survival, migration, angiogenesis, and carcinogenesis [5]. The most common types of *FGFR3* aberrations in UC are activating mutations, followed by gene rearrangements and amplification [6, 7]. *FGFR3* mutations are predominantly found in genetically stable UC [8], and have been associated with oncogenic progression in UC [9]. *FGFR3* gene rearrangements generate constitutively activated and oncogenic FGFR3 kinase protein products, and cellular dependence on these drivers confers sensitivity to selective FGFR inhibition [10, 11]. Furthermore, studies indicate that *FGFR3* mutation status could be used to guide anti-FGFR3 therapy [12]. However, previous molecular studies were performed mainly in patients with UBUC. Data on FGFR3 aberrations in the UTUC, particularly in the muscle invasive type, are not yet sufficient. Based on these considerations, this retrospective study aimed to evaluate the frequency and types of *FGFR3* gene aberrations in radically resected UC. We also compared the frequency of *FGFR3* alterations between UBUC and UTUC.

## Methods

### Patients

This study is a part of the Samsung Medical Center (SMC) Oncology Biomarker study (ClinicalTrials.gov identifier: NCT01831609). Tumor samples were collected from 74 consecutive patients with UC who underwent radical cystectomy or nephroureterectomy between 2012 and 2014, and had adequate specimen for molecular analysis. All patients provided written informed consent for the use of tumor tissues as well as their clinical data. This study was performed in accordance with the Declaration of Helsinki and approved by the Institutional Review Board of SMC (Seoul, Korea).

### Genomic DNA extraction

Our dedicated genitourinary pathologist (G.Y.K.) reviewed all pathology specimens to ensure the samples contained > 80% tumor cells with < 20% necrosis. Genomic DNA was extracted from the primary tumor tissues using a QIAamp DNA Mini Kit (Qiagen, Valencia, CA, USA). After extraction, we measured concentration as well as 260/280 and 260/230 nm ratio by spectrophotometer (ND1000, Nanodrop Technologies, Thermo-Fisher Scientific, MA, USA). Each sample was then quantified with the Qubit fluorometer (Life technologies, Carlsbad, CA, USA). Genomic DNA with > 10 ng measured by Qubit fluorometer was subjected to library preparation.

### DNA sequencing and copy number variations

We used the Ion Torrent Ampliseq™ cancer panel v2 to detect frequent somatic mutations that were selected based on a literature review. This panel examines 2855 mutations in 50 commonly mutated oncogenes and tumor suppressor genes (Additional file 1: Table S1). We constructed libraries using 10 ng of genomic DNA with the Ion AmpliSeq Library Kit and Ion Xpress Barcodes (Life Technologies). For barcoded library preparations, barcoded adapters from the Ion Xpress Barcode Adapters 1–96 Kit were substituted for the non-barcoded adapter mix in the Ion AmpliSeq Library Kit. Next, the multiplexed barcoded libraries were enriched by clonal amplification using emulsion polymerase chain reaction (PCR) on Ion Sphere Particles (Ion PGMTemplate 200 Kit) and loaded on an Ion 316 Chip. Massively parallel sequencing was carried out on an Ion PGM using the Ion PGM Sequencing 200 Kit v2. The primary filtering process was performed using Torrent Suite v3.6.0 and Ion Torrent Variant Caller v3.6 software. The pipeline included signaling processing, base calling, quality score assignment, adapter trimming, read alignment to 19 human genome references, mapping quality control, coverage analysis, and variant calling. For detection of copy number variations (CNV), nCounter Copy Number Variation CodeSets (NanoString Technologies, Seattle, WA, USA) were used with 300 ng of purified genomic DNA extracted from 2 to 3 sections of 4-μm-thick, formalin-fixed, paraffin-embedded (FFPE) representative tumor blocks using a QIAamp DNA FFPE Tissue Kit (Qiagen, Hilden, Germany). DNA was fragmented via AluI digestion and denatured at 95uC. Fragmented DNA was hybridized with the codeset of 257 genes (Additional file 2: Table S2) in the nCounter Cancer CN Assay Kit (Nanostring Technologies) for 18 h at 65uC and processed according to the manufacturer’s instructions. The nCounter Digital Analyzer counted and tabulated the signals of reporter probes.

### Bioinformatics and statistical analyses

We used cutoff values of greater than 6% variant frequency and more than 100X coverage to detect true mutational changes in accordance with previous reports and our own experience. Variant calls were further analyzed using the ANNOVAR, which included variant filtering and annotation using the Catalogue of Somatic Mutations in Cancer (COSMIC, http://cancer.sanger.ac.uk/cancergenome/projects/cosmic) database, dbSNP build 137, and amino acid change information. Variant calls from Ion AmpliSeq were further evaluated to reduce potential false-positives. Coverage (> 100X) and quality score (> 30) were considered as filtering criteria. For gene expression data from the NanoString nCounter assay, filtering of samples using quality control criteria was performed according to the manufacturer’s recommendations. All statistical analyses were performed by the Biostatistics and Clinical Epidemiology Center at our institute. The R for Windows v2.11.1 software (R Core Team, Vienna, Austria; http://www.r-project.org) was used for analysis of all data. We implemented the method found in the R “compound.Cox” package.

## Results

A total of 74 patients with primary tumor samples available were included: 34 patients for UTUC and 40 patients for UBUC (Table 1). Median age at the time of surgery of all patients was 64 years (range, 37 to 83). UC patients were predominantly male (86%), but the proportion of female patients was a bit higher in UTUC than in UBUC (26% vs. 8%, respectively). All but one UBUC had undergone lymph node dissection whereas it was performed in 53% of UTUC patients. In UBUC cohort, more than half of patients (65%) received neoadjuvant chemotherapy prior to radical cystectomy. In all patients, perioperative chemotherapy was a combination of gemcitabine plus either cisplatin or carboplatin, based on the patients’ renal function. There was no significant difference in other clinicopathological features including histology, tumor grade, pathological T (pT) stage, pathological N (pN) and lymphovascular invasion between UBUC and UTUC. Since all tumor samples were obtained at the time of radical surgery, the cohorts lacked early stage, superficial UC.

**Table 1:** Patient characteristics

	All patients (*n* = 74)	UTUC (*n* = 34)	UBUC (*n* = 40)
Age, years			
Median	64	65	64
Range	37 to 83	50 to 79	37 to 83
Gender			
Male	64 (86%)	25 (74%)	37 (93%)
Female	10 (14%)	9 (26%)	3 (8%)
pT			
1	3 (4%)	1 (3%)	2 (5%)
2	14 (19%)	5 (15%)	9 (23%)
3	55 (74%)	27 (79%)	28 (70%)
4	2 (3%)	1 (3%)	1 (3%)
pN			
0	11 (15%)	7 (21%)	4 (10%)
1	19 (26%)	4 (12%)	15 (38%)
2	23 (31%)	6 (18%)	17 (43%)
3	4 (5%)	1 (3%)	3 (8%)
Not evaluated	17 (23%)	16 (47%)	1 (3%)
Grade			
2	20 (27%)	11 (32%)	9 (23%)
3	54 (73%)	23 (68%)	31 (78%)
Lymphovascular invasion			
No	34 (46%)	15 (44%)	19 (48%)
Present	40 (54%)	19 (56%)	21 (53%)
Type of surgery			
Open	41 (55%)	15 (44%)	26 (65%)
Laparoscopic/robot-assisted	33 (45%)	19 (56%)	14 (35%)
Perioperative chemotherapy			
None	21 (28%)	10 (29%)	11 (28%)
Neoadjuvant	27 (36%)	1 (3%)	26 (65%)
Adjuvant	26 (35%)	23 (68%)	3 (8%)

*UTUC* Upper tract urothelial carcinoma, *UBUC* Urinary bladder urothelial carcinoma; *pT* pathological T stage, *pN* pathological N stage

Among 74 tumor samples tested, we found 16 (22%) actionable *FGFR3* gene aberrations. Table 2 presents the clinical and pathological characteristics of the 16 patients with *FGFR3* aberrations. In addition to 13 patients with *FGFR3* mutations, we identified three patients with translocation involving *FGFR3-TACC3* (Chr4) which was already considered a promising therapeutic target [13]. There was no significant difference in the frequency of *FGFR3* aberrations between UTUC (18%) and UBUC (25%) cohorts (*P* = 0.444). 31% of tumors with *FGFR3* aberrations were of grade 3 (i.e., poorly-differentiated, according to the WHO 1973 classification). Grade 3 and lymphovascular invasion were associated with a lower frequency of *FGFR3* aberrations (Table 3).

**Table 2:** Clinical and pathological characteristics of patients with *FGFR3* gene aberrations detected in surgical specimens

Patient	Age	Gender	Primary site	Pathologic stage	Grade	LVI	FGFR3
TCC_03	64	M	Bladder	pT3N0	3	No	FGFR3-TACC3 fusion
TCC_07	58	M	Bladder	pT2N0	2	No	Y373C
TCC_13	47	M	Bladder	pT2N2	2	Yes	R248C
TCC_14	61	M	Bladder	pT3N0	2	No	G370C
TCC_19	66	M	Bladder	pT3N0	2	No	746_747insG (NM_000142)
TCC_41	55	M	Renal pelvis	pT3Nx	2	No	FGFR3-TACC3 fusion
TCC_44	71	M	Renal pelvis	pT3Nx	3	Yes	N532D
TCC_48	50	M	Bladder	pT4N3	3	No	S249C
TCC_49	63	M	Bladder	pT1N2	2	Yes	A797P
TCC_50	59	F	Ureter	pT3N1	2	No	N532D
TCC_55	73	M	Bladder	pT2N0	2	No	S675S
TCC_56	54	F	Renal pelvis	pT3Nx	2	No	Y373C
TCC_61	78	M	Ureter	pT3Nx	3	No	Y373C
TCC_63	55	M	Ureter	pT3N2	3	Yes	R248C
TCC_70	66	M	Bladder	pT4N0	2	No	N532D
TCC_71	80	M	Bladder	pT2N0	2	No	FGFR3-TACC3 fusion

*FGFR3* Fibroblast growth factor receptor 3, *LVI* Lymphovascular invasion

**Table 3:** Rates of *FGFR3* gene aberrations according to patient characteristics

Characteristics	Total No.	FGFR3 aberration No.	*P* value
Primary site			0.444^a^
UBUC	40	10 (25%)	
UTUC	34	6 (18%)	
Gender			1.000^b^
Male	64	14 (22%)	
Female	10	2 (20%)	
Grade			< 0.001^b^
2	20	11(55%)	
3	54	5 (9%)	
Lymphovascular invasion			0.008^a^
No	34	12 (35%)	
Yes	40	4 (10%)	

^a^Chi-squared test

^b^Fisher’s exact test

**Fig. 1:**
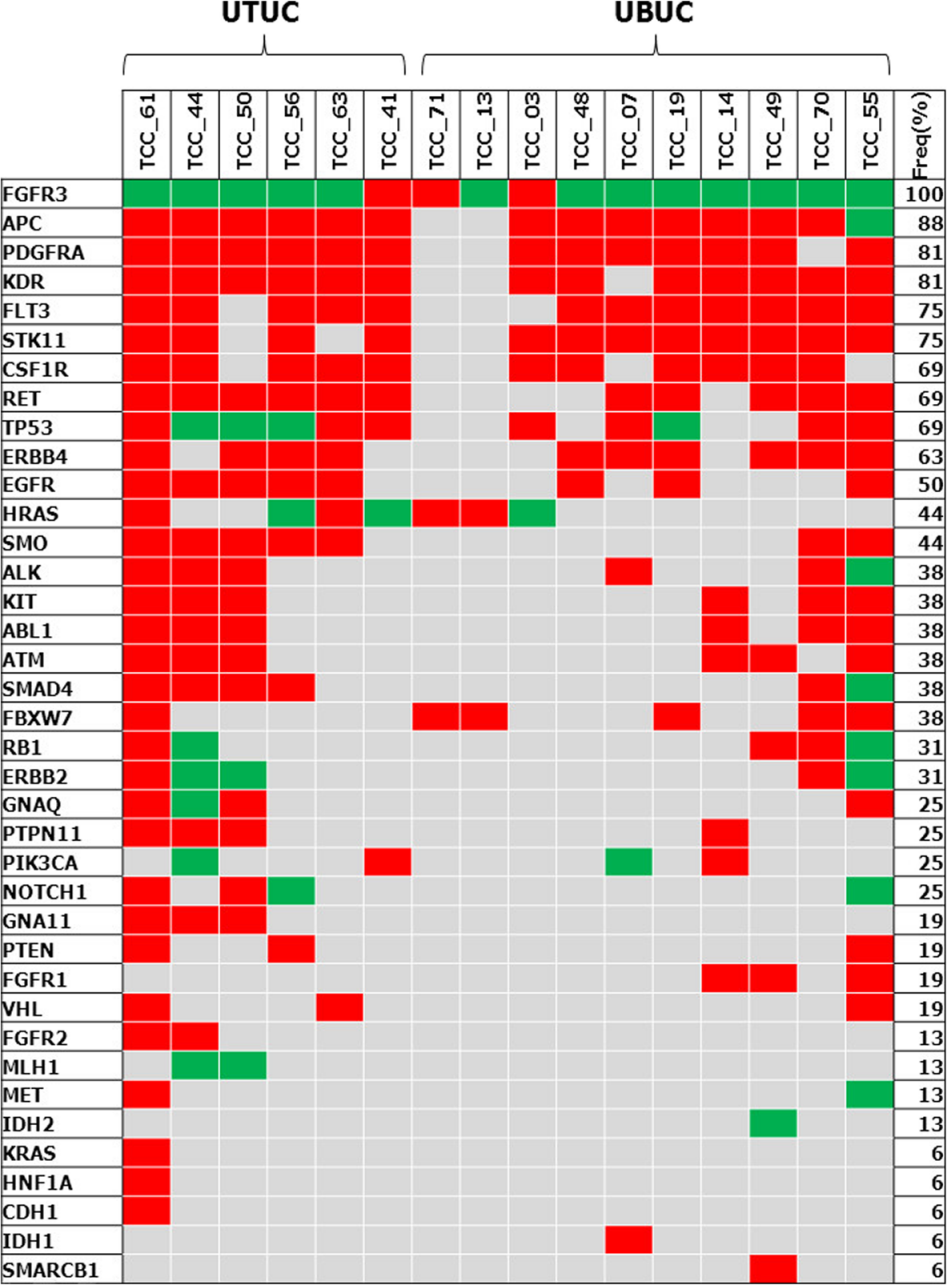
Distribution of additional mutations identified by Ampliseq (*n* = 16). Red squares indicate inactivating mutation. Green squares indicate activating mutation. *UTUC* Upper tract urothelial carcinoma, *UBUC* Urinary bladder urothelial carcinoma

We next investigated other genetic alterations in 16 patients with *FGFR3* gene aberrations (Fig. 1). As expected, we found no relevant differences in the incidence of both inactivating and activating mutations between UTUC and UBUC. The most frequently observed genetic mutation was *APC*, followed by *PDGFRA, KDR, FLT3,* and *STK11*. *HRAS* mutations were found in 7 patients. Interestingly, three of these *HRAS* mutations were found to be activating, actionable mutations (G12S, G13R and Q61R), unlike the previous study suggesting a mutual exclusion of *RAS* and *FGFR3* [14].

## Discussion

Radical cystectomy is the treatment of choice for muscle invasive UBUC [15], and radical nephroureterectomy is considered the standard treatment for UTUC [16]. However, the high rate of recurrence in these tumors necessitates novel approaches to systemic therapy. FGFR3 is considered a potential therapeutic target in UC, because recent studies show that FGFR3 activation is an important contributor to tumor development and angiogenesis in UC [17, 18]. Molecular tumor analysis and rational selection of patients are necessary in order to perform clinical trials involving FGFR-targeted agents. The present study demonstrated that *FGFR3* abnormalities are present in 22% of patients with UC who underwent radical resection. The majority of aberrations were *FGFR3* point mutations.

Although UTUC and UBUC have a similar histologic feature, there are epidemiologic and clinicopathologic differences between them [19]. Recently, several studies have reported molecular profiles of UTUC and UBUC [20–22], but controversy remains regarding whether UTUC is biologically distinct from UBUC. This is because, in part, of the relative rarity of UTUC hindering large-scale molecular studies. Our study focused on *FGFR3* aberrations in muscle-invasive UC and compared UTUC with UBUC. Compared to previous studies, relatively many cases of UTUB (*n* = 34) were included in the analysis and the results showed no significant difference in the frequency of *FGFR3* aberrations between UBUC (25%) and UTUC (18%). On the other hand, in a study comparing high-grade UTUC (*n* = 59) with UBUC (*n* = 102), overall landscape of genetic alterations was similar in both groups, although *FGFR3* were more frequently altered in UTUC than in UBUC (36% vs. 22%, respectively) [22]. In a comprehensive study of the genetics of UTUC, whole exome sequencing was performed in samples from 27 patients; *FGFR3* alteration was detected in 60% (9 of 15) of high-grade tumors and in 37.5% (3 of 8) of > pT2 tumors [21].

*FGFR3* mutations are common in low grade and early stage UCs, while they are less common in muscle-invasive tumors. In a previous meta-analysis for *FGFR3* mutations in UBUC, the frequency of *FGFR3* mutations decreased with increasing stage and grade: 65% in pTa, 30.2% in pT1, 11.5% in pT2–4 and 69.8% in G1, 68% in G2 and 18.6% in G3 [23]. The frequency of *FGFR3* mutations in the present study was infrequent with 18%; it is explained by that in our study, 96 and 73% had pT2–4 and G3 disease, respectively. We identified eight different mutations, including R248C, S249C, and Y373C, which consist more than 95% of mutations from radical cystectomy specimens in a previous study [12]. Preclinical models and early clinical trials suggest that these mutations have sensitivity to FGFR3 inhibitors [18, 24, 25]. Furthermore, we found four additional mutations (N532D, S676S, A797P, and 746_747insG) which have not been reported previously in the COSMIC database (accessed December 2017). Further studies are needed in order to evaluate if these mutations are pathogenic and represent valid targets for anti-FGFR3 therapy.

FGFR3 fusion proteins are additional type of mutational events in a subset of UCs with up-regulated FGFR3 expression. *FGFR3* fusions with *TACC3* and *BAIAP2L1* have been reported in UC cell lines and tissues [10, 18]. The clinical relevance of *FGFR3-TACC3* fusion in UC has been highlighted by results from preclinical and early clinical studies reporting promising responses to the treatment with FGFR inhibitors. In a phase I trial with FGFR inhibitor JNJ-42756493 (*n* = 65), five responses were observed; two of them harbored *FGFR3-TACC3* translocation [26]. It has been reported that the prevalence of *FGFR3-TACC3* fusion in UC ranged 2 to 6% [6, 22, 27]. As the majorities of studies analyzed samples from muscle-invasive cancer, the association between FGFR3 fusion and tumor grade or stage is still uncertain. In our study, *FGFR3-TACC3* translocation was observed in three patients (4%): one patient with high-grade tumor and two patients with low-grade tumor. On the other hand, Sfakianos et al. reported that all five *FGFR3-TACC3* translocations were detected only in high-grade UTUCs (*n* = 59) but in no low-grade tumors (0 of 23) [22].

Recent studies have reported encouraging data of FGFR3-targeted therapies in patients with advanced UC harboring *FGFR3* alterations. In a phase I expansion cohort study [28], 67 patients with *FGFR3-*altered UC were enrolled and treated with BGJ398, a selective FGFR1–3 inhibitor; 70.1% had received two or more systemic therapies. BGJ398 monotherapy was well tolerated and had response rate of 25.4% with a disease control rate of 64.2%. In a Phase II trial of erdafitinib [29], a pan-FGFR inhibitor, the 99 patients enrolled had a verified mutation in *FGFR3* (74.7%) or fusion in *FGFR2/FGFR3* (25.3%); 88.1% had received ≥1 line of prior systemic treatment. Erdafitinib showed a response rate of 40.4% and a disease control rate of 79.8%. Responses occurred in patients without prior exposure to chemotherapy (41.7%) as well as those previously treated with chemotherapy (40.2%). These results suggest that FGFR3-targeted therapies may represent a viable strategy for the treatment of *FGFR3*-altered UC in metastatic as well as perioperative settings.

Several limitations of our study warrant consideration. First, the results should be interpreted with caution given the limited number of patients and retrospective nature. Second, analysis using matched normal tissues was not performed. Third, the imbalance in the administration of neoadjuvant chemotherapy between UBUC and UTUC may affect the results, because it is known that neoadjuvant chemotherapy can induce mutational shift [30, 31]. Similarly, it should be noted that previous intravesical therapy may influence the results of mutational analysis in UBUC, although our study included only three patients who had received intravesical therapy.

## Conclusions

We report that *FGFR3* gene aberrations were detected in 22% of curatively-resected UC. The frequency was similar between UTUC and UBUC. Patients with *FGFR3* mutations or *FGFR3-TACC3* fusion may constitute potential candidates for a novel FGFR-targeted therapy in the perioperative setting. Further studies are warranted to reveal the functional significance of the *FGFR3* aberrations and better define subset of patients that benefit from anti-FGFR therapy.

## Additional files

Additional file 1: **Table S1.** List of targeted genes in Ion AmpliSeq™ Cancer Panel v2. (XLSX 79 kb)

Additional file 2: **Table S2.** Gene list of nCounter CodeSet. (XLSX 20 kb)

## Abbreviations

CNV: Copy number variation
COSMIC: Catalogue of somatic mutations in cancer
FFPE: Formalin-fixed, paraffin-embedded
FGFR3: Fibroblast growth factor receptor 3
PCR: Polymerase chain reaction
pN: Pathological N
pT: Pathological T
SMC: Samsung Medical Center
UBUC: Urinary bladder urothelial carcinoma
UC: Urothelial carcinoma
UTUC: Upper tract urothelial carcinoma
